# Modeling and Comparative Study on Cure Kinetics for CFRP: Autocatalytic vs. Neural Network vs. Angle Information-Enhanced RBF Models

**DOI:** 10.3390/polym17223059

**Published:** 2025-11-18

**Authors:** Xintong Wu, Linman Wei, Ming Zhang, Zhongling Liu, Bin Xiao, Xiaobo Yang, Zan Yang

**Affiliations:** 1 School of Advanced Manufacturing, Nanchang University, Nanchang 330031, China; wuxintong@ncu.edu.cn (X.W.); 13117806093@163.com (M.Z.); mp2513148534@163.com (B.X.); 2 Nanchang Jardine Advanced Composite Material Co., Ltd., Nanchang 330029, China; 3 College of Mechanical and Electrical Engineering, Lanzhou Jiaotong University, Lanzhou 730070, China; 4 State Key Laboratory of Precision Manufacturing for Extreme Service Performance, Central South University, Changsha 410083, China

**Keywords:** cure kinetics model, carbon fiber reinforced polymer, angle information-enhanced radial basis function

## Abstract

Carbon fiber reinforced polymer (CFRP) components require precise curing process control to ensure quality, but traditional phenomenological cure kinetics models face limitations in handling nonlinearity and data diversity. This study addresses the challenges in modeling the cure kinetics of carbon fiber reinforced polymer (CFRP) composites, where traditional phenomenological models lack generalizability and neural networks suffer from robustness issues due to their numerous hyperparameters and data dependency. To overcome these limitations, a novel machine learning model called the angle information-enhanced radial basis function (RBF) model is proposed, which integrates both Euclidean distance and angular relationships between data points to improve prediction stability and accuracy. The performance of this machine learning approach is systematically compared against an autocatalytic model and a neural network using dynamic DSC data from T700/2626 epoxy resin at multiple heating rates. The angle-enhanced RBF model balances accuracy, efficiency, and robustness, offering a reliable data-driven alternative for CFRP cure kinetics prediction without requiring extensive data or complex hyperparameter optimization, thus facilitating better process control in manufacturing.

## 1. Introduction

Carbon fiber reinforced polymer (CFRP) has been extensively utilized in various fields to replace conventional materials, owing to its excellent characteristics, such as higher specific strength and stiffness, design flexibility, and monolithic molding [1,2,3]. These characteristics make it particularly suitable for manufacturing critical aircraft components (e.g., wing skins, U-shaped flat tail leading edges, and tail structural components) that endure significant dynamic and vibratory loads during service [4,5]. In order to meet the strict requirements for size accuracy and quality of these components, they are usually manufactured using an autoclave process that involves hot-press curing of CFRP. During the autoclave process, CFRP is subjected to heating according to a predetermined thermal cure cycle while simultaneously being pressurized to a specified level [6]. This process involves a series of complex thermochemical and physical changes, including heat transfer, polymerization reaction, curing exotherm, and property evolution. These reactions may result in uneven distribution of temperature gradients and non-uniform distribution of degree of cure (DoC), which eventually leads to component deformation and quality deterioration. Among the numerous factors influencing the quality of CFRP components, the curing process and final curing state of resin play a critical role [7]. The curing of thermosetting resins is an irreversible process characterized by crosslinking and hardening, which progresses monotonically toward a fully cured state, and the response of the resin is often nonlinear. Therefore, modeling reliable cure kinetics of the resin in CFRP composites is indispensable for predicting material behavior, designing efficient processes, and controlling the performance quality of components [8,9].

To date, researchers have developed numerous modeling methodologies for cure kinetics. Cure kinetics models are generally categorized as either mechanistic or phenomenological models [10]. Mechanism models are derived from the equilibrium of various reactants involved in the complex reaction, which can make their development challenging. In comparison, phenomenological models describe the overall reaction rate without accounting for the complex details of the reactants and their reaction, offering a flexible, intuitive, and practical approach for studying cure kinetics across diverse formulations and curing conditions [11]. As a result, phenomenological models are widely adopted as the preferred framework for describing resin cure kinetics, with notable examples including the autocatalytic model, the n-order model, the Kamal–Sourour model, etc. Among them, the Kamal–Sourour model is the most widely studied model [12,13]. Lopez et al. [14] utilized the activation energy proposed by Kissinger to determine the parameters of the Kamal–Sourour model and successfully described the vulcanization process of silicone rubber. Hernandez-Ortiz et al. [15] developed a numerical methodology that fitted the autocatalytic Kamal–Sourour model exclusively to dynamic DSC data, bypassing traditional isothermal methods, and validated it on silicone rubber and phenolic resins. Sun et al. [16] focused on methods dealing with variable activation energy for epoxy-based systems; their investigation into autocatalytic behavior was conceptually aligned with the kinetic principles that the Kamal model described. Liu et al. [17] introduced a variable activation energy concept to account for ultrasonic non-thermal effects and incorporated this energy into the Sun–Gang modified autocatalytic equation.

However, Hardis and Muphalilele [18,19] demonstrated that the heat generated by the resin increases as the heating rate increases, including the initial curing temperature, peak curing temperature, and final curing temperature. Furthermore, the inherent discreteness and diversity of cure kinetics phenomenological models lead to difficulty in forming a unified and general model [20]. Due to the high nonlinearity of this inverse problem, determining the coefficients is challenging in the case of differential equations. Although researchers have recognized this issue and endeavored to predict cure kinetics through one-by-one fitting of experimental data, this approach does not permit continuous fitting of cure kinetics behavior. Consequently, it is unsuitable for continuous learning from the growing volume of resin cure kinetics data and hinders synchronous application of this knowledge in component manufacturing.

In recent years, alongside advances in big data and computational power, data-driven methods have demonstrated significant potential. Neural network models can learn the complex mappings inherent in cure kinetics in a black-box manner, providing novel approaches to handle the highly nonlinear problems encountered during the curing and molding process of CFRP [4,21,22]. Many researchers have reported that they have employed various neural network algorithms to predict problems related to cure kinetics. Carlone et al. [23] coupled an artificial neural network (ANN) with a finite element thermochemical model to optimize the thermal curing process of high-temperature composites. Fan et al. [24] demonstrated the application of a convolutional neural network (CNN) combined with the finite element (FE) method to predict process-induced deformation cloud maps of composite structures. Hui et al. [20] replaced conventional cure kinetics models with neural networks to comprehensively model the CFRP cure process. Yang et al. [4] developed lightweight neural networks with incremental learning capability that effectively addressed generalization and continuous learning challenges in cure kinetics modeling. Generally, the core of neural networks lies in stacking hidden layers or increasing the number of convolutional kernels to enhance their ability to learn essential features from cure kinetics data [25,26]. This inevitably necessitates the use of additional optimization operators, such as batch or stochastic gradient descent methods, to determine the values of their numerous hyperparameters. The large number of parameters, on the one hand, requires extra optimization methods to identify their optimal values; on the other hand, it demands a substantial amount of sample data to provide sufficient essential feature information for accurate parameter estimation. As a result, the specific outcomes of each prediction may vary significantly, indicating poor robustness, which may not fully meet the robustness requirements of the prediction results required by the cure kinetics model.

In contrast, classical machine learning models such as Gaussian Process (GP), Radial Basis Function (RBF), and Support Vector Machine (SVM) are typically derived from strict mathematical assumptions or principles to build mathematical models capable of fitting the core features of real cure kinetics data, and these models feature a rigorous derivation process and fewer hyperparameters [27,28,29]. Compared with neural networks, machine learning models provide stable prediction results and are less susceptible to the influence of hyperparameters. However, so far, almost no researchers have constructed accurate machine learning prediction models for curing behavior. Therefore, how to build a machine learning model that can balance predictive robustness and accuracy is the first research focus of this paper.

Moreover, although plenty of studies have reported that the curing behavior can be modeled through dynamic or isothermal processes utilizing various models and algorithms, the existing research results remain highly fragmented. Firstly, most studies are limited to the application and validation of a single model, lacking a systematic comparative analysis of phenomenological versus multiple data-driven models under a unified dataset and evaluation framework. Secondly, there is an insufficiently clear delineation of the relative strengths and weaknesses of different models regarding prediction accuracy, computational efficiency, and generalizability, which creates confusion for engineers in selecting appropriate models. Therefore, how to systematically experiment and analyze the machine learning model proposed in this paper with other models in terms of prediction accuracy, computational efficiency, and generalization ability is the second research focus.

Considering the above issues, this paper innovatively designed an angle information-enhanced RBF, in which both the angle information and Euclidean distance were simultaneously integrated, to improve the stability and accuracy for fitting the complex features in cure kinetics. Moreover, this work also focused on investigating the theoretical foundations of the existing cure kinetics modeling approaches—namely, the autocatalytic model and the neural network model—and conducted a comprehensive evaluation between the angle information-enhanced RBF and these models to validate the performance. Specifically, the dynamic heat flow of the resin was measured at various heating rates using differential scanning calorimetry (DSC), and the resulting data were integrated to serve as the foundation for subsequent cure kinetics modeling. In the developed network architecture, the degree of cure (DoC), temperature, and heating rate served as the input variables, while the reaction rate was the output; the entire dataset was utilized to train this model. Subsequently, the theoretical basis and model development process for the phenomenological neural network and the proposed machine learning model were examined. Finally, a comprehensive comparative evaluation was performed across multiple dimensions, including prediction accuracy, computational cost, and model robustness.

## 2. Materials and Experimental

T700/2626 epoxy composites are developed in the aerospace field. The 2626 epoxy resin used in this study was a thermoset and toughened epoxy resin, provided by Nanchang Jiading Company, Nanchang, China. The resins were frozen and stored at 253 K until use. The manufacturer’s recommended cure cycle is as follows: (1) Resin is heated from room temperature to 398 K at 2–3 K/min. (2) Temperature is held at 398 K for 120 min and finally cooled at 2 K/min.

To comprehensively study the thermomechanical behavior of 2626 epoxy resin, all DSC non-isothermal experiments were performed on a Netzsch DSC 3500 instrument, Selb, Bavaria, Germany (enthalpy accuracy: ±0.01%) [30]. Resin specimens weighing about 10 mg were sealed in aluminum hermetic pans and placed in the DSC for each measurement. The gas used in the experiment was a nitrogen atmosphere with a flow rate of 40 mL/min. Based on the cure characteristics of 2626 epoxy resin, the dynamic scans were ramped up from 293 K to 523 K at six various heating rates of 1.0, 2.5, 5.0, 10.0, 15.0, and 20.0 K/min, and the corresponding temperature and heat flow data during the process were monitored and used to calculate the degree of conversion rate.

## 3. Experimental Results

**Figure 1:**
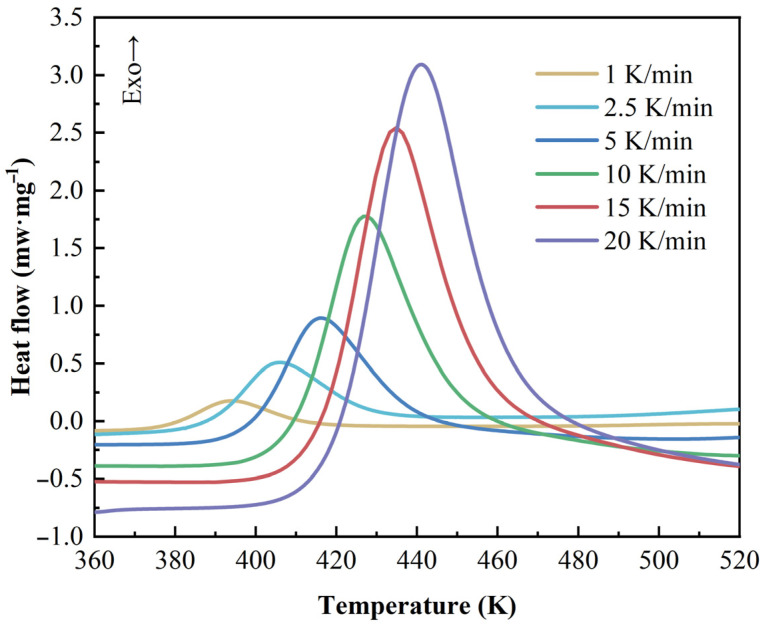
Dynamic DSC curves of epoxy resin at different heating rates (the ‘Exo’ arrow indicates the direction of an exothermic signal in the DSC curve).

As shown in Figure 1, from the dynamic scans, the normalized heat flow curves versus temperature can be obtained at different heating rates. With increasing temperature, the reaction is activated when the exothermic heat flux rate starts to deviate gradually from its steady-state value. The 2626 resin exhibits only one exothermic peak that shifts to a higher temperature with an increase in the heating rate. This phenomenon is mainly due to more external energy being applied to the resin within the relatively shorter total reaction time. The average amount of ultimate reaction heat per unit mass is calculated to be 156.62 J/g by the area under the curve. The results show that the 2626 resin releases energy mainly within a relatively long temperature range, and total exothermal energy is not that high.

**Figure 2:**
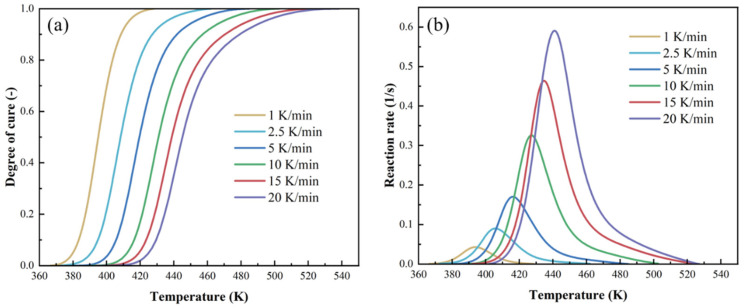
(**a**) Curves of the relationship between degree of cure and temperature at different heating rates; (**b**) curves of the relationship between reaction rate and temperature.

In Figure 2a, the degree of cure obtained from heat flow integration at different heating rates is plotted, and the curing reaction rate of the resin is presented in Figure 2b. The reaction mainly occurred between 370 K and 520 K. The curing curve demonstrates a tendency to shift toward higher temperatures with increasing heating rates. The conversion of resin proceeds as a sigmoidal curve at different heating rates. Furthermore, the temperature sensitivity of resin makes the interval between degree-of-cure (DoC) curves narrow at higher heating rates. Obviously, the reaction rate also increases as the heating rate rises.

## 4. Cure Kinetics Methodology

To ensure the accuracy and reliability of the determined kinetics, three distinct methods were adopted. This multi-faceted strategy allows for cross-validation and enhances confidence in the final estimated values. More details are given below.

### 4.1. Autocatalytic Model

The curing reaction of epoxy resin is exothermic, and DSC can monitor the heat flow signal during the curing process. The heat flow measured by DSC, denoted as dH/dt, represents the curing reaction rate [31]. This rate can be integrated to determine α, which indicates the degree of cure or conversion. The reaction rate is typically described by a temperature-dependent rate function *K*(*T*) and a conversion function *f*(*α*) as expressed in Equation (1):

(1) $$\frac{d\alpha }{dt}=K\left(T\right)f\left(\alpha \right)$$

where *dα/dt* is the cure kinetics rate, α is the curing degree of the resin, and *t* is the curing time. *K*(*T*) is the temperature relationship of the rate constant, and *f*(*α*) is the reaction mechanism function.

**Figure 3:**
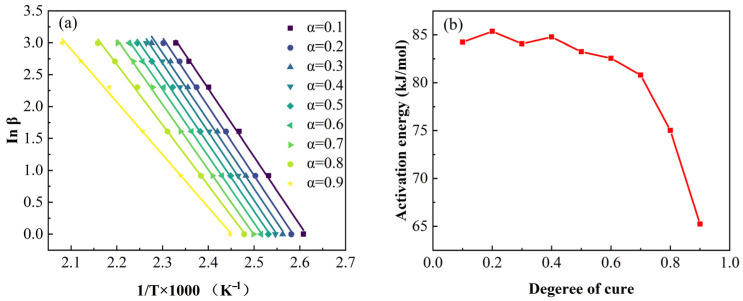
(**a**) FWO method fitting curves for resin at different degrees of cure; (**b**) variation in resin activation energy with degree of cure.

For epoxy resin, the phenomenological model is the most prevalent approach for predicting the cure kinetics behavior, typically based on fitting empirical equations. The crucial step for cure kinetics modeling is to understand the reaction progression for selecting the appropriate reaction mechanism function. These models can principally be classified into two major types: nth order and autocatalytic. As shown in Figure 3, the heat evolution of 2626 resin reaches its maximum at 30–40% of the reaction progress, demonstrating cure kinetics behavior characteristic of an autocatalytic reaction. The autocatalytic model uses a single rate constant to simulate the entire curing process. In practice, multiple events may lead to highly complex mechanisms. Therefore, using multiple rate constants can yield more accurate modeling outcomes. The autocatalytic model, a phenomenological model that incorporates two rate constants, has been widely used to model numerous resin systems [32]. Equation (2) can be expressed as follows:

(2) $$\frac{d\alpha }{dt}=K\left(T\right)f\left(\alpha \right)=Aexp\left(-\frac{E_{\alpha }}{RT}\right)\alpha^{m}{\left(1-\alpha \right)}^{n}$$

Therein, *A* is the pre-exponential factor, *E_α_* is the activation energy, *R* is the ideal gas constant, and *T* is the absolute temperature, and *m* and *n* represent the reaction orders.

Another critically important parameter is the apparent activation energy *E_α_*. In non-isothermal curing processes with constant heating rate *β*, these parameters can be determined through the isoconversional method and single-speed method. Given that isoconversional methods provide theoretically rigorous results, the Flynn–Wall–Ozawa (FWO) isoconversional method was employed to calculate both *E_α_* and the pre-exponential factor *A* [33]. The FWO method’s Equation (3) is as follows:

(3) $$\log\beta =\log\frac{AE_{\alpha }}{g\left(\alpha \right)R}-\frac{0.4567E_{\alpha }}{RT}-2.315$$

where *β* is the heating rate, and *g*(*α*) is the integral function of conversion.

**Figure 4:**
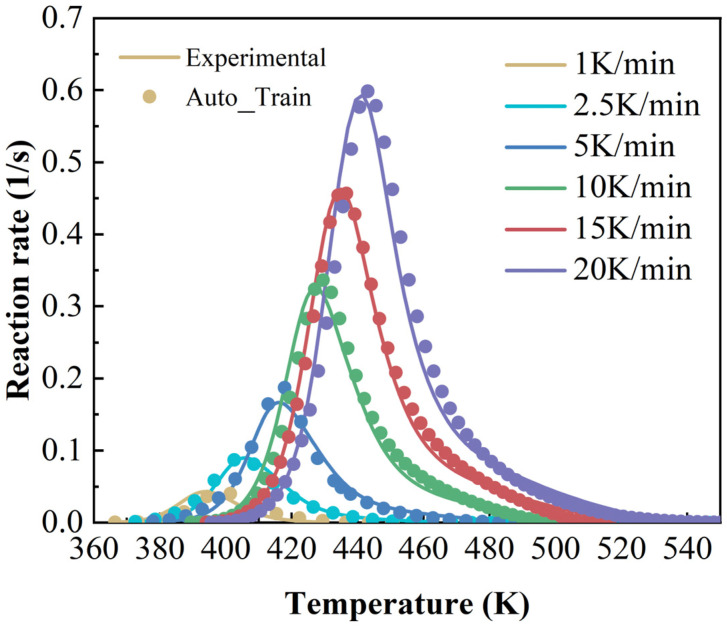
Comparison curves of experimental and calculated values of dα/dT~T at different heating rates (autocatalytic model).

In the FWO model, when selecting a fixed degree of cure α, the integral form of the kinetic mechanism function *g*(*α*) remains constant. If the curve of ln*β* versus 1/Tα (where Tα is the temperature at the selected conversion α) is plotted for different heating rates, the apparent activation energy *E_α_* can be determined from the slope of the linear fit. As shown in Figure 4, the corresponding linear fitting curves and the variation in the activation energy (*E_α_*) as a function of the degree of cure (α). Analysis revealed a significant change in activation energy when the degree of cure exceeded 0.4. Given the substantial variation in activation energy during the curing process, the conventional constant *E_α_* model proves inadequate. The modified Sun–Gang variable activation energy framework was consequently employed to characterize the cure kinetics of 2626 epoxy resin [34]. This model assumes that the activation energy *E_α_* and pre-exponential factor *lnA* are functions of α (degree of cure). Equation (4) can be expressed as follows:

(4) $$\beta \frac{d\alpha }{dT}=exp\left(p_{1}+p_{2}\alpha +p_{3}\alpha^{2}+p_{4}\alpha^{3}\right)exp(-\frac{p_{5}+p_{6}\alpha +p_{7}\alpha^{2}+p_{8}\alpha^{3}}{RT})\alpha^{m}{\left(1-\alpha \right)}^{n}$$

Through polynomial fitting, the values of each parameter can be determined. Taking *α* (degree of cure) and *T* (temperature) as independent variables, and *β·dα/dT* (heating rate multiplied by the rate of cure conversion) as the dependent variable, the model parameters were regressed using the Particle Swarm Optimization (PSO) algorithm [35] to obtain the global optimal solution, as listed in Table 1.

The accuracy of the cure kinetics equation obtained using the modified Sun–Gang model can be verified by comparing the experimentally derived and model-calculated dα/dT~T curves. The comparison curves are shown in Figure 4 (Auto_train means autocatalytic model—training).

### 4.2. Neural Network Model

**Figure 5:**
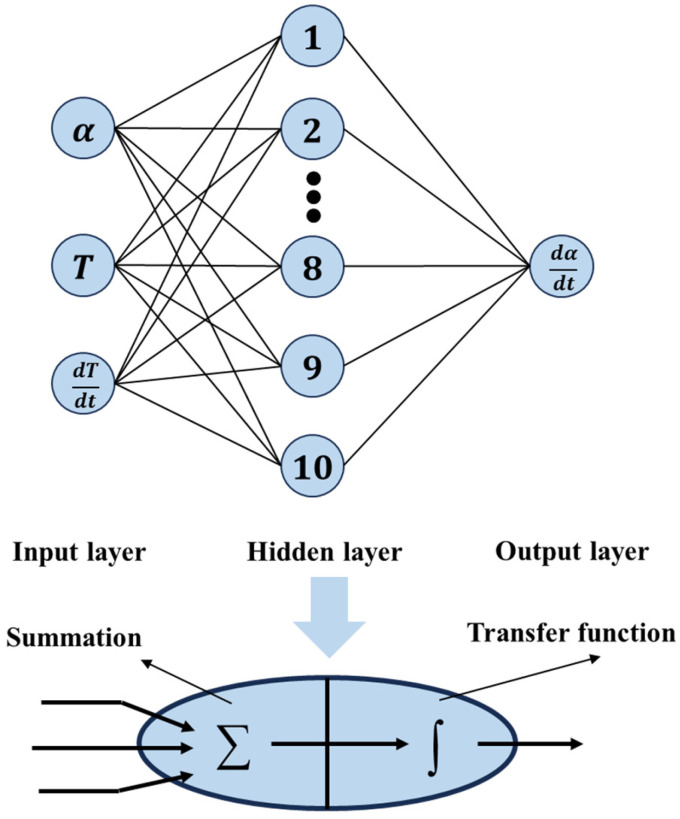
The structure of the proposed BP neural network.

The artificial neural network (ANN) is a biologically inspired computational system composed of interconnected processing units (neurons) organized in layered architectures. The backpropagation neural network (BPNN) represents a prominent subclass of ANN [36,37]. BPNN has two core attributes: (1) a strictly feedforward multilayer architecture including input, hidden, and output layers, and (2) an error-driven learning algorithm called backpropagation. This error-driven learning mechanism enables BPNN to iteratively refine synaptic weights by propagating output discrepancies backward through the network, making it exceptionally effective for modeling complex relationships in tasks like non-isothermal DSC data prediction. In non-isothermal DSC analysis of resins, BPNN employs a layered architecture where the input layer receives experimental parameters, one or more hidden layers perform nonlinear transformational processing, and the output layer generates critical predictions. Figure 5 illustrates schematically the concepts of BPNN.

Equation (5) serves to describe the BPNN model [38]:

(5) $$\left\{\begin{array}{c} z_{j}=f\left(\sum_{i=1}^{m}w_{ij}x_{i}+b_{j}\right)\\ y_{k}=f\left(\sum_{j=1}^{n}w_{jk}z_{j}+b_{k}\right) \end{array}\right.$$

where *x_i_* represents the input layer; *z_j_* represents the hidden layer; *y_k_* represents the output layer; *w_ij_* and *w_jk_* are weights, where the weight between the *i* neuron in the input layer and the *j* neuron in the hidden layer is represented by *w_ij_*, and the weight between the *j* neuron in the hidden layer and the *k* neuron in the output layer is represented by *w_jk_*; and *b_j_* and *b_k_* are biases. Each neuron processes the inputs using weight factors *w_ij_* and biases *b_j_* to produce an output result. Every neuron estimates the output signal by computing the sum of weighted inputs and biases, then transforming this sum through a sigmoid transfer, as shown in Equation (6):

(6) $$f\left(x\right)=\frac{1}{1+e^{-x}}$$

Any BPNN must be trained using available input/output datasets before being applied to practical problems. During training, the network first processes inputs forward through weighted connections and a transfer function to generate predictions, then calculates prediction errors against experimental DSC data. These errors are backpropagated through the network layers, where gradients for each weight are computed using the chain rule of calculus, and then updated via optimization methods to minimize the loss. Based on the cure kinetics results, a BPNN with a three-layer structure was designed, comprising an input layer, a hidden layer, and an output layer. Each layer contains multiple neurons composed of a fully connected adder and activation function. Three factors serve as the input, and one factor serves as the output. Empirical formulas and refereed literature revealed that the BPNN model adequately meets training requirements when configured with 10 nodes for each hidden layer. The inputs to the network are the curing degree α, temperature T, and heating rate dT/dt. The output is the curing reaction rate. The dataset used was a series of cure cycle data obtained from the previous DSC test. This dataset was randomly divided into three subsets: training (70%), validation (15%), and testing (15%). The network was trained using the Levenberg–Marquardt backpropagation algorithm, with mean squared error (MSE) as the optimization objective. Training terminated when the MSE reached the target threshold of ≤0.001. The training model implementation result in Matlab R2025a was as follows: After 506 training iterations, the MSE meets the preset error requirements, with a total runtime of 1.57 s. After multiple training iterations, the network converges to an optimized function that can provide effective responses to any similar inputs. Upon completion of training, the model must be validated using the test dataset. Validation data was employed to assess the generalization capability of the network prediction and guard against overfitting.

**Figure 6:**
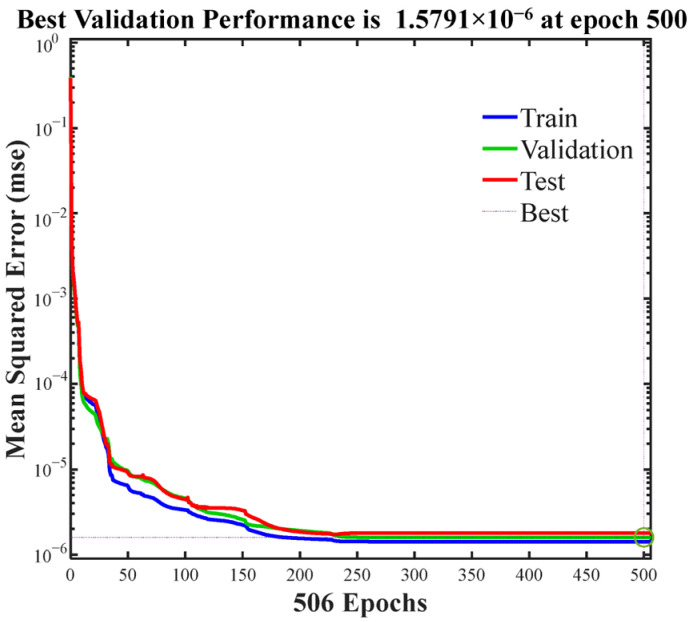
Convergence performance of the NN model.

**Figure 7:**
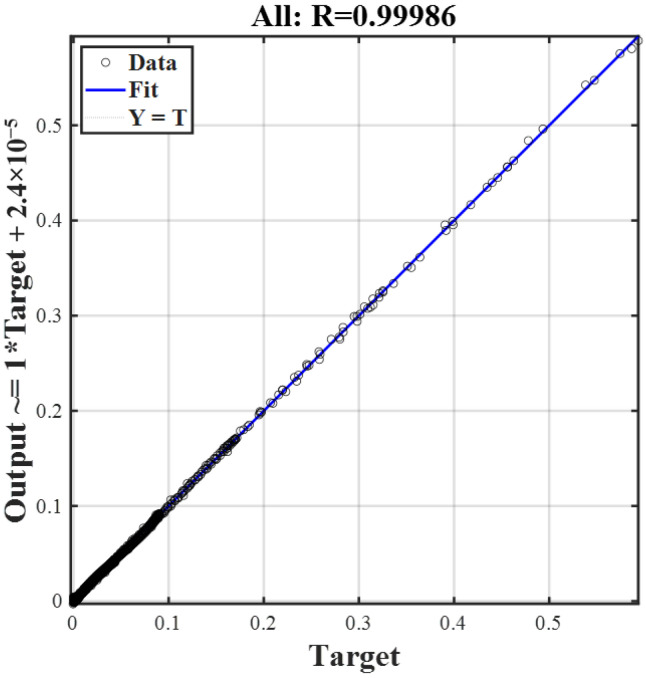
Multiple regression analysis of the NN model.

The cure kinetics are typically highly nonlinear. The multilayer perceptron architecture of BP neural networks, coupled with its activation functions, makes it particularly well-suited to handle this complexity. As shown in Figure 6, the maximum training epochs is set to 1000, and the actual training ends in the 506th epoch. The loss function curve exhibits a sharp decline during the initial phase of training and subsequently plateaus in the later stages. It can be concluded that the training process has reached convergence and has thus come to an end. The mean square error (MSE) of the validation data indicates that the training process effectively avoided overfitting. Furthermore, a multiple regression analysis is conducted to evaluate the goodness of fit, as illustrated in Figure 7. The coefficient of determination R reaches a value of 0.99986 across all datasets, indicating excellent agreement between the experimental data and the predictions generated by BPNN. Therefore, the trained model can be confidently utilized for further validation and practical applications.

**Figure 8:**
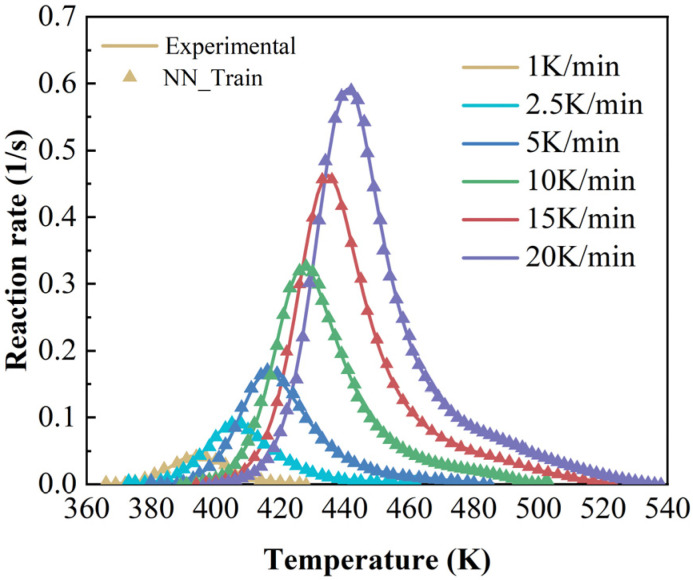
Comparison curves of experimental and calculated values of dα/dT~T at different heating rates (neural network model).

The accuracy of the cure kinetics equations derived from the BPNN model can be verified by comparing the experimentally derived dα/dT~T curves with those calculated by the model. The comparative curves are presented in Figure 8.

### 4.3. Machine Learning Model

Radial Basis Function (RBF) has been widely adopted as a machine learning model in many real-world engineering problems due to its exceptional balance between computational efficiency and predictive accuracy [39,40]. Existing implementations predominantly employ the conventional RBF architectures, particularly those utilizing cubic kernels, for machine learning modeling. These traditional approaches construct basis functions exclusively through Euclidean distance metrics between center points and sample data, inherently limiting correlation representation to scalar distance information. In fact, pure Euclidean distance fails to capture the intrinsic angular relationships between data points. Inspired by polar coordinate systems, an angle information-enhanced radial basis function is constructed, which synergistically integrates both angular positional information (quantified through angle values) and Euclidean distance metrics, thereby establishing a more comprehensive geometric representation. This dual-information framework significantly enhances the model’s capacity to characterize complex optimization landscapes by preserving both magnitude and directional relationships between solution candidates.

**Figure 9:**
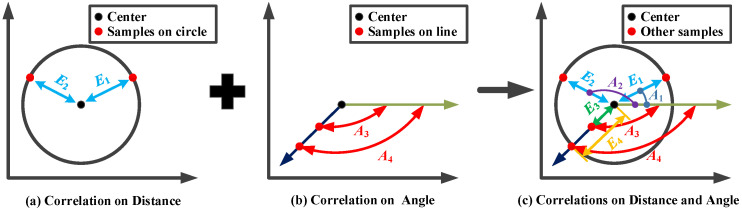
Illustration of correlation calculated by three different approaches.

Figure 9 illustrates the correlation calculated by three different approaches. If the correlation is measured only based on Euclidean distance in Figure 9a, the built RBF cannot distinguish these samples on a circle centered on the center point; thus, the predicted values will be unreliable. By contrast, as shown in Figure 9b, if the RBF is constructed only based on the angle information-based correlation, such a model cannot provide accurate prediction when these samples are all on the same straight line as the center point. Hence, we incorporated both the angle information and Euclidean distance information to formulate the basis functions, as shown in Figure 9c. Then, the constructed RBF can better adapt to complex landscapes to provide high accuracy.

For *β* samples, i.e., $T=\left(t_{1},\ldots ,t_{\beta }\right)\subseteq R^{n}$ and the objective values $F_{\beta \times 1}={\left(f_{1},\ldots ,f_{\beta }\right)}^{T}$, the angle information-enhanced RBF may be expressed via Equation (7):

(7) $${\hat{f}}^{RBF}(t)=\sum_{i=1}^{n}\varepsilon_{i}\delta \left(\left\|t-t_{i}\right\|\right)+\sum_{i=1}^{n}\gamma_{i}\kappa \left(Cosine(t,t_{i}\right))+Poly(t)$$

where ${\hat{f}}^{RBF}(t)$ donates the RBF prediction; $\delta \left(\left\|t-t_{i}\right\|\right)$ and $\kappa \left(Cosine\left(t,t_{i}\right)\right)$ are the basis functions measured based on Euclidean distance and angle information, respectively; $t_{i}$ is the *i*-th center; $\varepsilon_{i}$ and $\gamma_{i}$ are the *i*-th weights of the weight vectors such as $\varepsilon ={\left(\varepsilon_{1},\ldots ,\varepsilon_{n}\right)}^{T}$ and $\beta ={\left(\beta_{1},\ldots ,\beta_{n}\right)}^{T}$ for both basis functions, respectively; and Poly(t) donates the first-order linear polynomial. Thus, the indefinite equation system can be rewritten in matrix form, as shown in Equation (8):

(8) $$E_{\beta \times \beta }\cdot \varepsilon_{\beta \times 1}+A_{\beta \times \beta }\cdot \gamma_{\beta \times 1}+Poly_{\beta \times (n+1)}\cdot D_{(n+1)\times 1}=F_{\beta \times 1}$$

In Equation (8), *E_β×β_ and A_β×β_* are the matrices calculated by Euclidean distance-based and angle information-based basis functions, respectively, i.e., $E_{i.j}=\delta \left(\left\|t_{i}-t_{j}\right\|\right)$ and $A_{i.j}=\kappa \left(\left\|t_{i}-t_{j}\right\|\right)$; $D_{(n+1)\times 1}$ donates the weight vector for *Poly*(*t*); and *Poly_β×(n+1)_* represents the vector of the Poly(t) for the input matrix T. Then, we expand Equation (8) to determine all the parameters as follows:

(9) $$\left(\begin{array}{ccc} E_{\beta \times \beta } & A_{\beta \times \beta } & Poly_{\beta \times (n+1)}\\ Poly_{(n+1)\times \beta }^{T} & 0_{(n+1)\times \beta } & 0_{\left(n+1)\times (n+1\right)}\\ 0_{(n+1)\times \beta } & Poly_{(n+1)\times \beta }^{T} & 0_{\left(n+1)\times (n+1\right)} \end{array}\right)\left(\begin{array}{c} \varepsilon_{\beta \times 1}\\ \gamma_{\beta \times 1}\\ D_{(n+1)\times 1} \end{array}\right)=\left(\begin{array}{c} F_{\beta \times 1}\\ 0_{(n+1)\times 1}\\ 0_{(n+1)\times 1} \end{array}\right)$$

Then, the equation system in Equation (9) can be expressed as Equation (10) below:

(10) $$H_{\left(\beta +2n+2)\times (2\beta +n+1\right)}{Μ}_{(2\beta +n+1)\times 1}={Ζ}_{(\beta +2n+2)\times 1}$$

Thus, all the parameters can be derived theoretically based on Equation (11).

(11) $$\begin{array}{cc} {Μ}_{(2\beta +n+1)\times 1}= & {\left(H_{\left(2\beta +n+1)\times (\beta +2n+2\right)}^{T}\cdot H_{\left(\beta +2n+2)\times (2\beta +n+1\right)}\right)}^{-1}\\ & \cdot H_{\left(2\beta +n+1)\times (\beta +2n+2\right)}^{T}\cdot {Ζ}_{(\beta +2n+2)\times 1} \end{array}$$

In Equation (11), *H^T^* donates the transpose of *H*, and *M_(_*_2*β+n+*1*)×*1_ contains all the derived parameters. Therefore, the angle information-enhanced RBF is rigorously derived through mathematical theory, which means that the designed RBF with the same modeling data have uniqueness.

**Figure 10:**
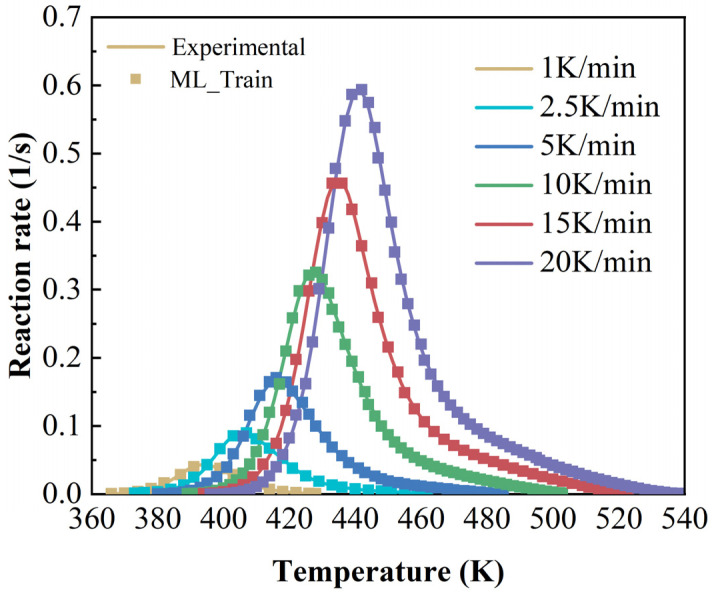
Comparison curves of experimental and calculated values of dα/dT~T at different heating rates (machine learning model).

The accuracy of the cure kinetics equations derived from the angle information-enhanced RBF model can be verified by comparing the experimentally derived dα/dT~T curves with those calculated by the model. The comparative curves are presented in Figure 10.

## 5. Discussion

**Figure 11:**
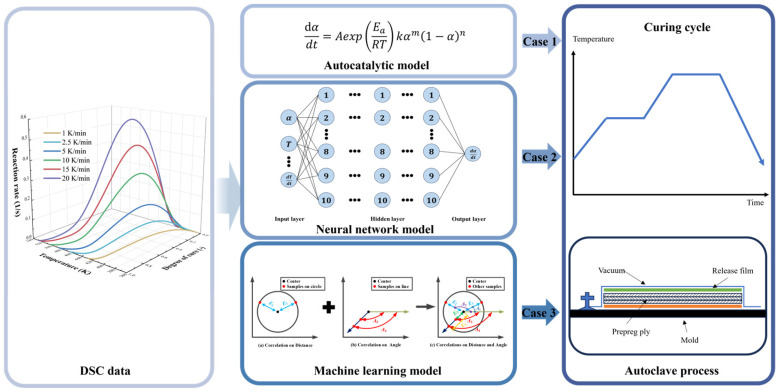
The operation conditions of the three methods and their application framework in the autoclave process.

**Figure 12:**
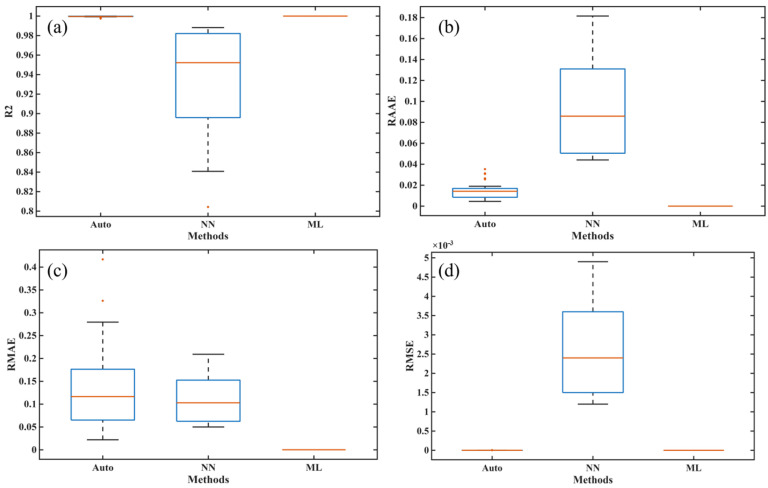
The training effects of different models on the DSC dataset: (**a**) R2, (**b**) RAAE, (**c**) RMAE, and (**d**) RMSE.

**Figure 13:**
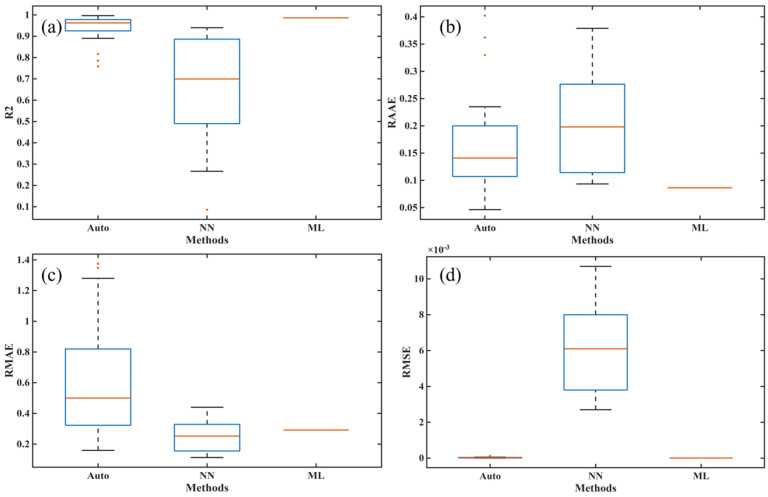
The fitting effects of different models on the DSC dataset: (**a**) R2, (**b**) RAAE, (**c**) RMAE, and (**d**) RMSE.

**Figure 14:**
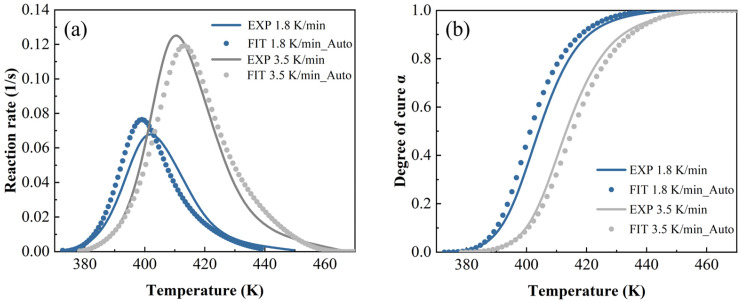
The autocatalytic model predictions at a heating rate of 1.8 K/min and 3.5 K/min: (**a**) reaction rate and (**b**) DoC.

**Figure 15:**
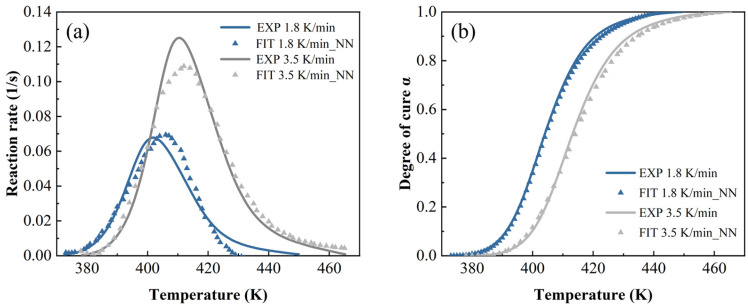
The neural network model predictions at a heating rate of 1.8 K/min and 3.5 K/min: (**a**) reaction rate and (**b**) DoC.

**Figure 16:**
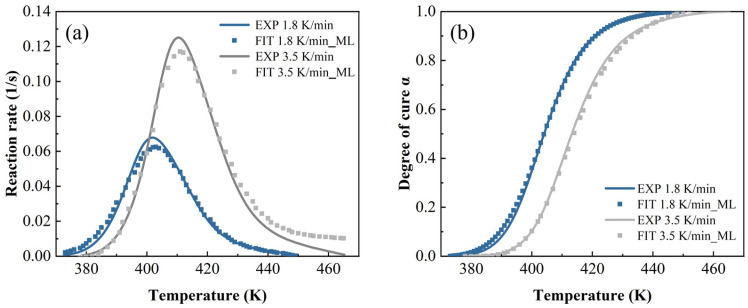
The machine learning model predictions at a heating rate of 1.8 K/min and 3.5 K/min: (**a**) reaction rate and (**b**) DoC.

In order to fully verify the robustness and ensure the fairness of the comparison, we independently ran the training and prediction processes of each algorithm 30 times, and we also calculated the training and prediction time of each algorithm in the same hardware and software environments. Specifically, the training and prediction of NN (neural network model) and ML (machine learning model) were performed on a laptop with a CPU with CPU@13900HX (2.2 GHz, 24 cores, 32 threads) and 16 GB of memory using Matlab R2025a. Furthermore, to ensure the correctness of the NN program, we used the built-in net function in the Deep Learning Toolbox of Matlab R2025a to train the NN. Figure 11 presents the operation of the three methods and the architecture diagrams for their application in the autoclave process. Table 2 and Table 3 show the performance of all comparison methods in five metrics, including four core metrics widely used to validate model accuracy, such as R2, RAAE, RMAE, and RMSE, as well as a time metric, where Time1 and Time2 represent the time taken for the methods to complete the training and prediction processes, respectively. Figure 12 and Figure 13 show the box plots corresponding to the four accuracy indicators obtained by three methods in 30 runs during the training and prediction processes. Figure 14, Figure 15 and Figure 16 present the results of a single run during the prediction process.

In Table 2, it can be observed that the mean values of all metrics obtained by Auto (autocatalysis approach) are worse than those of both NN and ML models, which directly means that Auto performed worst in terms of accuracy and efficiency during the training process. Moreover, ML’s performance is better than NN in terms of mean values for four accuracy metrics, indicating that ML has higher accuracy. The Std values obtained by ML are zero, indicating that the results are completely consistent after multiple runs, which is consistent with the rigorous theoretical derivation of ML given in Section 4.3. Similar results can also be seen in Table 3. Thus, the proposed ML not only shows the best performance in terms of training and predicting accuracies but also provides the strongest robustness.

As shown in Figure 12 and Figure 13, the training and fitting effects of both NN and Auto are also worse than those of ML. Furthermore, the Auto has outliers in both the training and prediction stages, and the NN also has outliers in the prediction process, indicating significant instability in the results obtained from multiple runs of these two methods. The reason the NN performs unstably is that the process of determining its many hyperparameters through optimizers such as batch or stochastic gradient descent has randomness. Also, the instability of the autocatalytic model lies in its highly coupled and complex nonlinear model, and solving its parameters is an ill-posed inverse problem that heavily relies on the performance of optimization algorithms and initial conditions.

In Figure 14, Figure 15 and Figure 16, both the autocatalytic and NN have biases in predicting peak size and temperature occurrence, while NN only has slight biases in predicting peak size. This indicates that ML is more accurate in predicting trends throughout the entire process. By contrast, when the heating rate is high and the temperature is high, ML’s prediction is slightly worse than other methods.

In summary, compared to Auto and NN, ML has stronger robustness and higher prediction accuracy because it does not involve complex optimization operators to determine hyperparameters. Considering that ML models can maintain consistent results in multiple runs, designing more accurate ML models based on the characteristics of cure kinetics data may be one of the important directions worth studying in the future.

## 6. Conclusions

This study addresses the limitations of traditional modeling methods in CFRP cure kinetics by proposing an angle information-enhanced radial basis function (RBF) model and conducting a systematic comparative analysis with autocatalytic and neural network models. Based on the comprehensive comparative analysis of autocatalytic, neural network, and angle information-enhanced RBF models for predicting the cure kinetics of T700/2626 epoxy resin, it can be conclusively determined that the proposed angle information-enhanced RBF model demonstrates superior performance. The model achieved perfect training accuracy (R2 = 1.000) with zero standard deviation across 30 independent runs, demonstrating exceptional robustness. Its prediction accuracy (R2 = 0.986) significantly outperformed both the neural network (R2 = 0.941) and autocatalytic (R2 = 0.666) models while maintaining computational efficiency, with training and prediction times of 1.79 s and 0.151 s, respectively. The neural network, despite reasonable accuracy, showed variability (Std = 0.0602) due to its optimization-dependent hyperparameters, while the autocatalytic model suffered from both inaccuracy and instability (Std = 0.230). The RBF model’s minimal prediction errors (RMSE = 6.90 × 10^−6^) and consistent performance across all heating rates confirm its reliability. This research validates that the angle information-enhanced RBF model provides a high-performance data-driven alternative for CFRP cure kinetics prediction. It not only improves the precision of tracking thermochemical changes during curing but also facilitates the design of more efficient autoclave processes and better control of CFRP component quality. In the future, this method will be applied to various epoxy resin systems to comprehensively evaluate the universality of this model.

## Figures and Tables

**Table 1:** Modified Sun–Gang model kinetic parameters.

A (min^−1^)	Energy (KJ/mol)	Order of Reaction	Correlation Coefficient
P1 = 14.74	P5 = 53.54	m = 0.565	R^2^ = 0.98
P2 = 3.90	P6 = 9.62		
P3 = −1.41	P7 = 0.58	n = 1.023	
P4 = −0.05	P8 = 3.89		

**Table 2:** Comparison of three methods on five metrics for training data.

Methods	R2		RAAE		RMAE		RMSE		Time 1
	Mean	Std	Mean	Std	Mean	Std	Mean	Std	Mean
ML	1.00 × 10^0^	0.00 × 10^0^	1.64 × 10^−5^	0.00 × 10^0^	1.29 × 10^−4^	0.00 × 10^0^	2.07 × 10^−12^	0.00 × 10^0^	1.79 × 10^0^
NN	9.99 × 10^−1^	5.93 × 10^−4^	1.49 × 10^−2^	8.04 × 10^−3^	1.38 × 10^−1^	9.18 × 10^−2^	1.44 × 10^−6^	1.56 × 10^−6^	6.30 × 10^−1^
Auto	9.33 × 10^−1^	5.44 × 10^−2^	9.38 × 10^−2^	4.31 × 10^−2^	1.11 × 10^−1^	4.93 × 10^−2^	2.60 × 10^−3^	1.14 × 10^−3^	9.47 × 10^−3^

**Table 3:** Comparison of three methods on five metrics for testing data.

Methods	R2		RAAE		RMAE		RMSE		Time 2
	Mean	Std	Mean	Std	Mean	Std	Mean	Std	Mean
ML	9.86 × 10^−1^	0.00 × 10^0^	8.63 × 10^−2^	0.00 × 10^0^	2.92 × 10^−1^	0.00 × 10^0^	6.90 × 10^−6^	0.00 × 10^0^	1.51 × 10^−1^
NN	9.41 × 10^−1^	6.02 × 10^−2^	1.63 × 10^−1^	8.44 × 10^−2^	6.12 × 10^−1^	3.47 × 10^−1^	2.97 × 10^−5^	3.04 × 10^−5^	4.23 × 10^−3^
Auto	6.66 × 10^−1^	2.30 × 10^−1^	2.02 × 10^−1^	8.20 × 10^−2^	2.49 × 10^−1^	9.21 × 10^−2^	6.04 × 10^−3^	2.23 × 10^−3^	3.88 × 10^2^

## Data Availability

The original contributions presented in this study are included in the article. Further inquiries can be directed to the corresponding author.

## Abbreviations

The following abbreviations are used in this manuscript:

CFRP	Carbon fiber reinforced polymer
RBF	Radial basis function
DoC	Degree of cure
ANN	Artificial neural network
CNN	Convolutional neural network
FE	Finite element
GP	Gaussian Process
SVM	Support Vector Machine
DSC	Differential scanning calorimetry
FWO	Flynn–Wall–Ozawa
PSO	Particle Swarm Optimization
BPNN	Backpropagation neural network
MSE	Mean squared error
NN	Neural network model
ML	Machine learning model
